# Effect of oral silymarin on liver function in pediatric acute lymphoblastic leukemia in the maintenance phase: a double-blind randomized clinical trial

**DOI:** 10.3389/fphar.2024.1295816

**Published:** 2024-01-12

**Authors:** Aziz Eghbali, Mahnaz Sadeghian, Ali Ghasemi, Roghayeh Rahimi Afzal, Aygin Eghbali, Kazem Ghaffari

**Affiliations:** ^1^ Clinical Research Development Center of Aliasghar Hospital, Iran University of Medical Sciences, Tehran, Iran; ^2^ Department of Pediatrics, Iran University of Medical Sciences, Tehran, Iran; ^3^ Department of Biochemistry and Hematology, Faculty of Medicine, Semnan University of Medical Sciences, Semnan, Iran; ^4^ Department of Pediatrics, Amir Kabir Hospital, Arak University of Medical Sciences, Arak, Iran; ^5^ School of Medicine, Iran University of Medical Sciences, Tehran, Iran; ^6^ Student Research Committee, Khomein University of Medical Sciences, Khomein, Iran; ^7^ Department of Basic and Laboratory Sciences, Khomein University of Medical Sciences, Khomein, Iran

**Keywords:** acute lymphoblastic leukemia, pediatrics, liver function tests, silymarin, liver toxicities

## Abstract

**Introduction:** Liver dysfunction is one of the most common disorders in patients with acute lymphoblastic leukemia (ALL). In recent studies, silymarin has been observed to have hepatic protective effects. Therefore, in this study, the effect of oral silymarin on the hepatic functions of patients with ALL was investigated.

**Methods:** In the present double-blind clinical trial study, 121 patients with ALL over 5 years of age were divided into two groups after obtaining informed consent. The subjects were randomly divided into a silymarin-treatment group and a placebo group. In the silymarin-treatment group, patients received 70 mg oral capsules of silymarin twice daily or syrup of silymarin three times a day (each 5 ml of syrup contains 50 mg of silymarin). Patients were examined once a month for 9 months to receive capsules and measure the levels of alanine aminotransferase (ALT), aspartate transferase (AST), alkaline phosphatase (ALP), gamma-glutamyl transferase (GGT), bilirubin, albumin, and cholesterol.

**Results:** Comparison of changes before and after treatment in the two groups showed that receiving oral silymarin resulted in a slight significant decrease in the levels of ALT, AST, GGT, and bilirubin (*p* < 0.05), but had no effect on ALP, albumin, and cholesterol (*p* > 0.05).

**Discussion:** The results of the present study showed that in pediatric patients with ALL, silymarin intake improves liver function. The very strong antioxidant effect of silymarin may explain its protective effect on the liver.

**Clinical Trial Registration**: IRCT20150119020715N10.

## Introduction

Cancer is one of the main causes of death in developed and developing countries and usually has a sudden onset. Cancer in childhood and adolescence usually occurs from birth to 19 years of age and is considered a life-threatening disease (Seiler and Jenewein, 2019). About 25% of the diagnosed cancers are related to pediatric acute lymphoblastic leukemia (ALL); thus, ALL is the most common pediatric cancer. The peak age of ALL incidence is between 2 and 5 years old, and its annual prevalence is 36.2 per 1 million people (Inaba and Pui, 2021). Hematological malignancies in Iran are among the sixth-most common malignancies in both sexes (Dastgiri et al., 2011).

Chemotherapy alone or in combination with other treatment approaches is still considered the treatment of ALL (Sheykhhasan et al., 2022). In the treatment of children with ALL, the administration of chemotherapy drugs is often associated with hepatotoxicity (Ladas et al., 2010). For this reason, the administration of chemotherapy drugs is stopped, especially during the maintenance phase of treatment. In a study, it was reported that more than half of patients with ALL experience grade 2 or more hepatotoxicities during the maintenance treatment phase (Farrow et al., 1997).

On the other hand, stopping the use of chemotherapy drugs will increase the risk of bone marrow relapse (Matutes et al., 1991; Schmiegelow, 1991; Ladas et al., 2010; Fehér and Lengyel, 2012). Among chemotherapy drugs, methotrexate and 6-mercaptopurine are used in the maintenance treatment of ALL, and both drugs are associated with hepatotoxicity. Also, different degrees of liver fibrosis have been observed after long-term use of these chemotherapy drugs (Farrow et al., 1997). Until today, there have been no adjunctive agents that can protect the liver and maintain liver function against the continuation of chemotherapy. Therefore, the need for a hepatoprotective drug is felt to implement the optimal doses of chemotherapy without the need to reduce the recommended doses, thereby increasing the survival of children with ALL.

Silymarin is derived from Milk thistle plant and contains various flavonolignans such as silybin, silychristin, silydianin, and 2,3-dehydroderivatives of silymarin flavonolignans. These materials have strong antioxidant, membrane stabilization, and anti-inflammatory properties, as well as restorative and proliferative properties (Polyak et al., 2010; ElSayed et al., 2019).

Considering that liver disorders and subsequently the increase of liver enzymes are one of the most common disorders in patients with leukemia treated with chemotherapy agents (Meir et al., 2001; Shahriari et al., 2020), this study aimed to investigate the effect of oral silymarin on liver enzymes in pediatric patients with ALL undergoing chemotherapy referred to Amirkabir Arak Hospital.

## Materials and methods

### Study subjects

**FIGURE 1:**
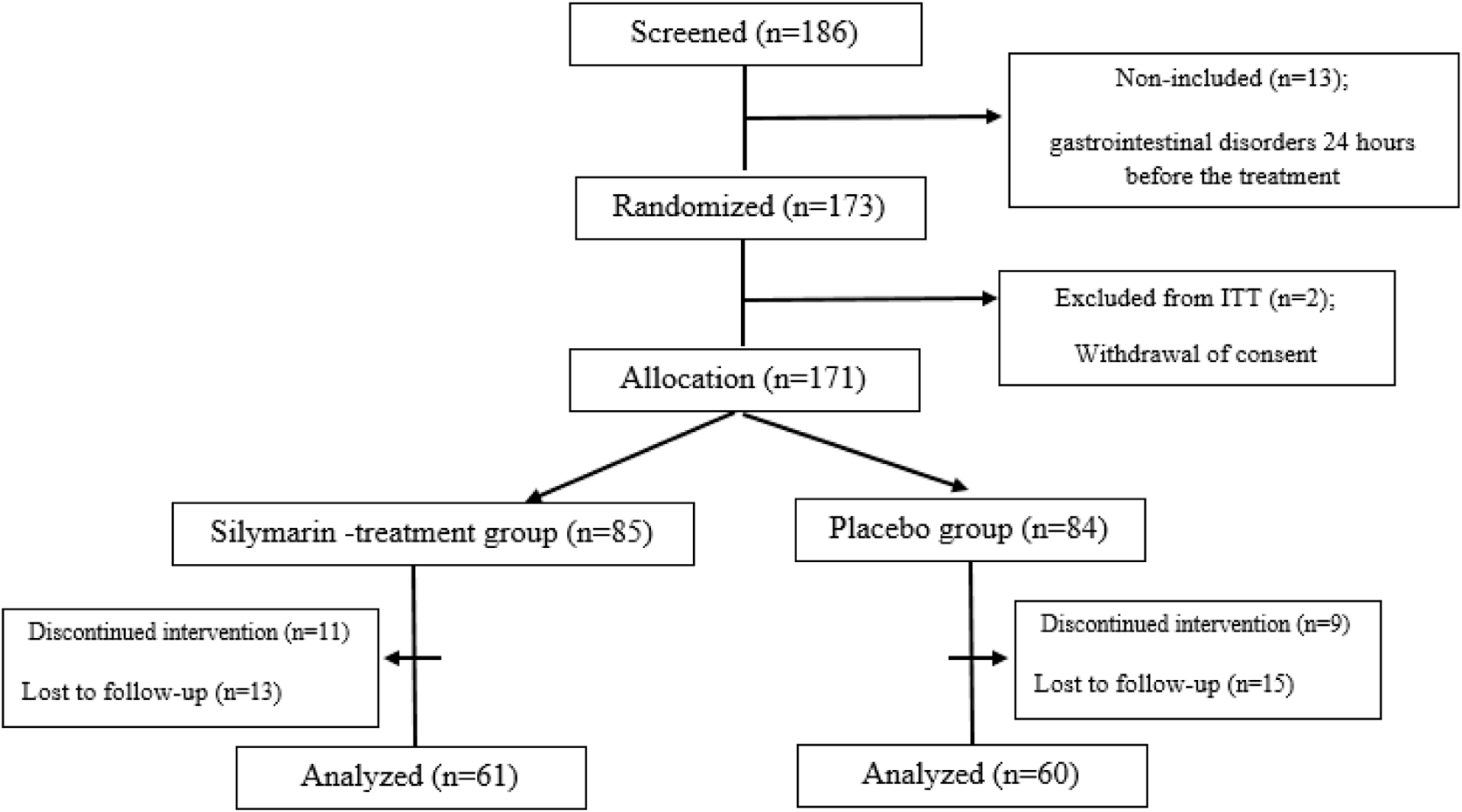
Flow chart of study procedure. ITT; intent-to-treat population.

This double-blind, randomized study was conducted from May 2020 to June 2022. A total of 173 patients over 5 years old with ALL in the maintenance phase were randomized in this study at Amir Kabir Hospital, Arak, Iran, considering 20% dropouts in each group. At the beginning of the study, a checklist was provided in which information on demographic and basic clinical data of all patients, such as the age, alanine aminotransferase (ALT), aspartate transferase (AST), alkaline phosphatase (ALP), gamma-glutamyl transferase (GGT), bilirubin, albumin, and cholesterol, were extracted. The sample size was calculated based on a study power of 80% with a type one error (α) of 5% and a statistical significance (α) of 95% (*p* = 0.05) using SPSS 25.0 software (SPSS, Inc., Chicago, IL, United States). Randomization (allocation ratio 1:1) was done by a biostatistician based on a simple randomization method using a computerized random number table inside the clinic. In this way, the patients were randomly divided into a silymarin-treatment group and a placebo group. The flowchart of the study is shown in Figure 1.

### Inclusion and exclusion criteria

Inclusion criteria included patients over 5 years with leukemia in the maintenance phase who were referred to Amir Kabir Hospital in Arak, cases that were satisfied with participating in the study, and patients who had no history of other hematologic and liver diseases such as HBV, HCV, HIV, and impaired liver enzymes (AST and ALT greater than or equal to 60, total cholesterol above 200, bilirubin more than or equal to 2, diabetes, smoking, hypertension, cardiovascular diseases, cholecystectomy, active infection, and GGT more than or equal to 60). We also excluded patients who were no longer willing to stay and collaborate in the study.

### Study intervention

In the silymarin-treatment group, patients received 70 mg oral capsules of silymarin (prepared by Ponik Pharmaceutical Company, Isfahan, Iran) twice daily (every 12 h) or syrup of silymarin (prepared by Zardband Pharmaceutical Company, Tehran, Iran) in case of inability to swallow the capsule form, three times a day, every 8 h (each 5 mL of syrup contains 50 mg Silymarin).

For the control group, placebo tablets and syrup (prepared by Ponik Pharmaceutical Company, Isfahan, Iran) were similar to silymarin in terms of dosage, shape and color. Silymarin and placebo were labeled by nurses with blue and red markers, respectively, so researchers and patients were blinded to treatment allocation until the end of the study. Then, based on the randomization program, the nurse distributed colored capsules to the patients by assigning an identification code to each patient. Parents were asked to be careful when taking medicine and not to stop taking medicine for any reason without consulting a doctor. Parents were also asked to record the number of supplements taken to determine adherence to treatment. Patients were treated for a period of 6 months, and every month, assessments of the liver enzymes mentioned above were performed, and the information was recorded.

### Statistical analysis

Data were expressed as mean ± SD for numerical variables. The two groups were compared using Pearsonʼs χ^2^ test for categorical variables. Mean levels of serum AST, ALP, bilirubin, and GGT before and after intervention were compared within groups using the independent sample *t*-test. Statistical analyses were performed using SPSS 25.0 software (Inc., Chicago, IL, United States) and a genetic analyzer (ABI PRISM 310, Applied Biosystems). *p* < 0.05 was considered statistically significant.

## Results

A total of 121 patients (61 patients in the silymarin-treatment group and 60 patients in the placebo group) completed the study, as presented in the CONSORT diagram (Figure 1). Most of the patients included in the study had an elevated ALT (77.7% of patients). In total, 45 patients (37.2%) in the silymarin group and 49 patients (40.5%) in the placebo group showed an increase in ALT. Also, 79% of patients in the silymarin-treatment group and 84% of patients in the placebo group experienced hepatotoxicity. Hepatotoxicity was observed in grades 2 and 3, and in grade 4, hepatotoxicity was not observed.

The mean age (±SD) in the silymarin-treatment group and placebo group was 8.6 ± 0.5 and 8.1 ± 0.3 years, respectively. The minimum and maximum ages in the silymarin and placebo groups were 5–14 and 6–13 years, respectively (Table 1). The comparison of the two groups showed that there was no significant difference between the two groups in terms of age (*p* = 0.451). In other words, the two groups were similar in terms of age. In total, 67 patients (55.4%) were male and 54 (44.6%) were female (Table 1). The study of the two groups using the Pearsonʼs χ2 test and Student’s *t*-test showed that there was no statistically significant difference between the two groups in terms of gender, weight, height, body mass index, ALT, AST, ALP, GGT, bilirubin, albumin and cholesterol (*p* > 0.05).

**TABLE 1:** Demographic characteristics and baseline clinical parameters of patients.

Characteristics	Silymarin group (N = 61)	Placebo group (N = 60)	*p*_value_
Mean age ±SD, yrs	8.6 ± 0.5	8.1 ± 0.3	0.932^b^
Min-Max	5–14	6–13	
Gender, n (%)			0.935^a^
Male	34 (55.7)	33 (55.0)	
Female	27 (44.2)	27 (45.0)	
Mean weight ±SD, Kg	27.3 ± 17.7	26.3 ± 16.8	0.886^b^
Mean height ±SD, Cm	129.5 ± 17.6	131.1 ± 18.7	0.357^b^
Mean body mass index ±SD, Kg/m2	12.6 ± 8.2	13.1 ± 10.1	0.321^b^
Mean AST ±SD, (IU/L)	142.6 ± 23.5	141.5 ± 20.9	0.327^b^
Mean ALT ±SD, (IU/L)	180.0 ± 21.8	181.5 ± 19.8	0.695^b^
Mean ALP ±SD, (IU/L)	245.1 ± 151.2	242.9 ± 158.4	0.443^b^
Mean GGT ±SD, (IU/L)	68.1 ± 15.6	69.9 ± 21.4	0.557^b^
Mean bilirubin ±SD, μmol/L	10.5 ± 11.8	10.6 ± 12.3	0.927^b^
Mean albumin ±SD, g/L	45.3 ± 11.3	44.6 ± 12.1	0.459^b^
Mean cholesterol ±SD, mg/dL	186 ± 51.8	182 ± 58.1	0.667^b^

SD; standard of deviation, n; number, ALT; alanine aminotransferase, AST; aspartate transferase, ALP; alkaline phosphatase, GGT; gamma-glutamyl transferase.

^a^ Pearsonʼs χ2 test was used.

^b^ Student t-test was used. Normal values: ALT <49 IU/L, AST <35 IU/L, ALP <104 IU/L, GGT <32 IU/L, albumin 35–50 g/L, bilirubin <17 μmol/L, and cholesterol less than 200 mg/dL.

Counting the number of capsules consumed showed that patients consumed more than 93.5% of the capsules, which shows the level of adherence to the treatment. In general, all patients tolerated the capsules well. However, parents reported mild gastrointestinal symptoms in several patients, but none of the complications were serious and resolved spontaneously after a few days.

**FIGURE 2:**
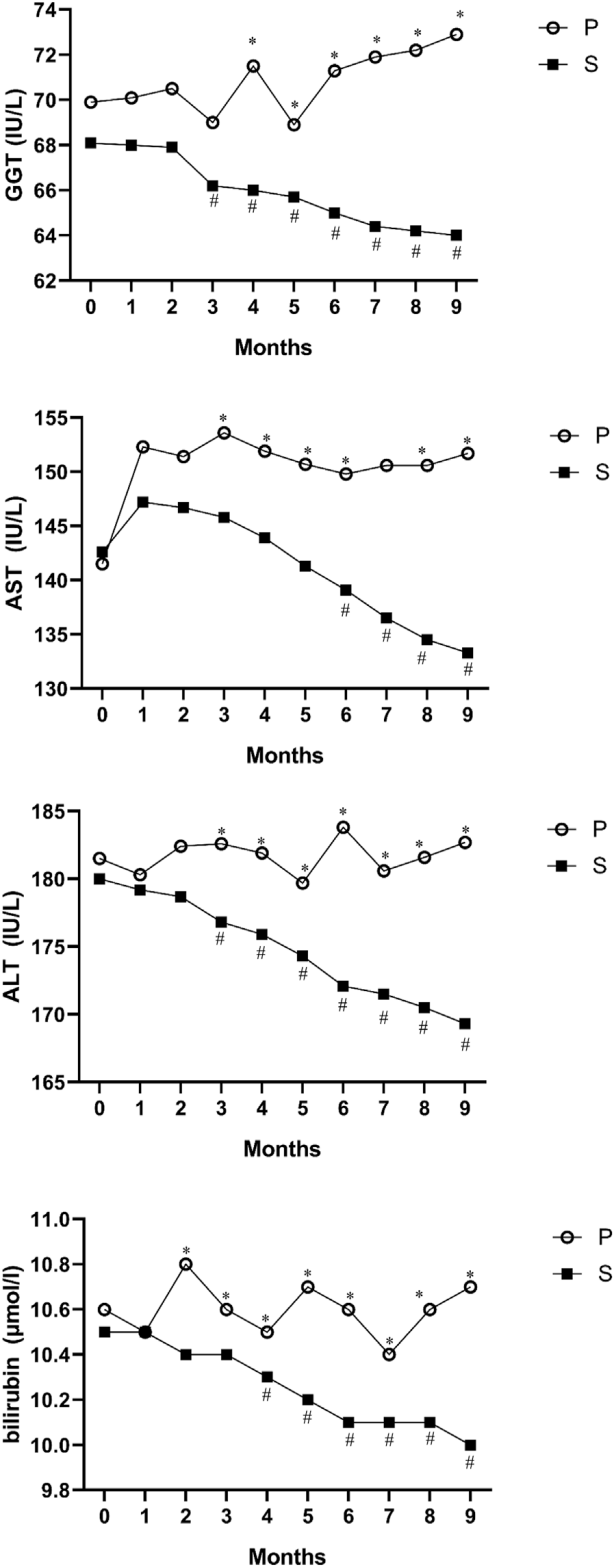
Mean values of ALT, AST, GGT and bilirubin before and after intervention in the studied groups. ALT; alanine aminotransferase, AST; aspartate transferase, and GGT; gamma-glutamyl transferase. *Significant difference in the comparison between the silymarin-treated group and the placebo group for each month point (point 0 vs. 0 to point 9 vs. 9), # significant difference in the comparison between different points of the month and the baseline point (point 1 vs. 0 to point 9 vs. 0) in the silymarin treatment group.

The hepatoprotective effect of silymarin was documented by a slight significant decrease in serum levels of ALT, AST, GGT and bilirubin in the silymarin-treatment group compared with the placebo group in ALL patients (*p* < 0.05, Figure 2).

There were no significant differences in ALP, albumin, and cholesterol levels at baseline or at the end of the treatments in either group (data not shown).

The mean dose of each potentially modifiable chemotherapeutic agent for hepatotoxicity administered during the intervention period in the silymarin and placebo groups was as follows: Vincristine, silymarin 1.8 mg/dose; placebo, 1.5 mg/dose; Methotrexate, silymarin 25 mg/m^2^/week; placebo, 20 mg/m^2^/week; and 6 mercaptopurine, silymarin 500 mg/m^2^/week; placebo, 413 mg/m^2^/week.

We assessed the mean reductions in intraindividual ALT, AST, ALP, GGT, bilirubin, albumin and cholesterol over 135 and 270 days from baseline by using difference scores. No significant differences between the silymarin-treatment group and placebo in ALP, albumin, or cholesterol from baseline to month 9 were observed (*p* > 0.05). However, in the silymarin-treatment group, mean reductions of ALT, AST, GGT, and bilirubin from baseline (day 0) to day 135 (mean reductions of 4.2, 1.6, 2.3, and 0.3, respectively) and from baseline (day 0) to day 270 had significant reductions (mean reductions of 12.2 and 10.8, 4.1, and 0.6, respectively).

## Discussion

Leukemia is one of the most common childhood malignancies, affecting approximately 40 children per million people under the age of 29 (Howlader et al., 2013). Various studies show a high incidence of hepatotoxicity in leukemia patients undergoing treatment (Christ et al., 2018; Denton et al., 2018). Mild liver dysfunction, especially elevated ALT, is common in children and adults with ALL (Baillargeon et al., 2005). Segal et al. (Segal et al., 2010) conducted a study on 147 children with ALL between 2001 and 2006. Similar to the findings of our study, they found that elevated levels of liver transaminase and hyperbilirubinemia were common manifestations in children with ALL.

This indicates the importance of finding a medication to control hepatotoxicity in pediatric with ALL. Livergel tablets contain Milk Thistle extract. This plant is one of the most effective and useful medicinal plants for liver disease (Behrman and Vaughan, 1983). This medicinal plant is more than 2000 years old in botanical medicine. The main ingredient in Milk thistle, which is the main ingredient in Livergel and protects the liver, is called silymarin, which helps regenerate liver cells damaged by alcohol and toxins. It also prevents the entry of various toxins into the cells by altering the outer layer of hepatic cells (Moradveisi et al., 2017). In addition, Silymarin further stimulates the liver to secrete bile, which helps purify the liver as well as improve gastrointestinal function. Silymarin also has the unique property of protecting the liver, and to date, no man-made chemical drug has demonstrated similar properties. For example, consumption of amanita, which grows in forests, causes poisoning and serious damage to the liver, and despite advances in pharmacology, the use of silymarin is still considered the best treatment option in these cases (Matutes et al., 1991).

This is a clinical study to investigate the effect of oral silymarin on liver enzymes in pediatric patients with ALL undergoing chemotherapy in the maintenance phase. We found that silymarin can be administered to children in the maintenance phase of treatment for ALL. The administration of a 9-month course of silymarin was associated with a slight significant decrease in ALT, AST, GGT, and bilirubin. No chemotherapy dose reductions, unusual toxicities, or treatment delays were observed during silymarin treatment.

Consistent with our results, Ladas et al. (Ladas et al., 2010), in their study to evaluate the safety and feasibility of silymarin over a 28- and 56-Day period for the treatment of hepatotoxicity in children with ALL who were receiving maintenance-phase chemotherapy, reported that their studied patients did not show statistically significant changes in mean ALT, AST, and bilirubin at Day 28, but at Day 56, the silymarin-treatment group had a significantly lower AST and a trend toward a significantly lower ALT. Differences in results may be due to different doses or durations of silymarin intake (long-term effects of the supplement).

In a study conducted in 2016 by Famouri et al. (Famouri et al., 2016), the effect of silymarin on non-alcoholic fatty liver in children was investigated. In this randomized cross-sectional clinical trial, 40 children with non-alcoholic fatty liver were divided into two treatment groups. Patients in the first group received the silymarin capsule at a dose of 140 mg three times a day, and after a 1-month cleansing period, they received a placebo for 3 months. Patients in the second group initially received a placebo, and then they were treated with silymarin. Liver enzymes were measured at the beginning of the study and after the first and second stages of the research. At the end of the 3 months, the patients underwent an ultrasound again. Their results indicated that silymarin reduces the degree of fatty liver in children with non-alcoholic fatty liver.

Also, in 2012, a review study was conducted by Fehér et al*.* (Fehér and Lengyel, 2012) to investigate the use of silymarin in the prevention and treatment of hepatic diseases, and it was concluded that silymarin is a safe and effective antioxidant drug in animal models. However, further studies on human samples are needed to confirm the effectiveness of this supplement.

The results obtained in this study showed that receiving oral silymarin in a 9-month treatment period leads to a slight significant decrease in serum levels of AST, ALT, bilirubin, and GGT in children with leukemia, but no significant effect was observed on cholesterol or albumin. In other words, silymarin seems to have protective effects on the liver in children with ALL.

In a case report by A. McBride et al*.* (McBride et al., 2012), the effects of milk thistle on the control and treatment of hepatotoxicity were evaluated in a patient with acute myeloid leukemia who had elevated liver enzymes following chemotherapy and did not respond to supportive therapies. They found that milk thistle had potential protective effects on the liver during chemotherapy. Their findings were similar to the results of the present study, which demonstrated that oral silymarin reduced liver enzyme levels in children with ALL and had protective effects on the liver.

In a review study conducted by Hadi et al. (Hadi et al., 2019), the effects of silymarin supplements on the metabolic status of patients with diabetes were evaluated, and the results showed that silymarin has beneficial effects on the metabolic status and oxidative stress of patients with diabetes. Also, a review study by Vahabzadeh et al*.* (Vahabzadeh et al., 2018) and a study by Ebrahimpour et al. (Ebrahimpour-Koujan et al., 2018) showed the effects of silymarin on reducing insulin resistance. One of the reasons for this is that since inflammation and oxidative stress contribute to insulin resistance, lipid peroxidation, and cardiovascular disease, reducing inflammatory biomarkers, indicators of oxidative stress, and hyperlipidemia can help better control diabetes.

After analyzing the clinical literature, it is clear that there is a considerable variation in the recommended dosage and duration of treatment. It is important to mention that herbal or nutritional supplements were not frequently included in phase 1 studies when examining the impacts on supportive care for this clinical trial. Therefore, we chose a short course of treatment and a conservative dose. New research on men with prostate cancer indicates that a higher dose of 13 g per day should be considered for future trials, suggesting that our current dosage may have been overly cautious. In our patient population, Phase 1 trials play a critical role in identifying the best dose and duration for preventing and treating hepatic toxicity.

However, there were limitations in the present study. Liver enzymes cannot accurately estimate chemotherapy-induced liver cell damage. Therefore, it is suggested that the protective effect of drugs on hepatotoxicity is best measured by liver biopsy or non-invasive tests such as liver fibroscan.

## Conclusion

The results of the present study showed that in pediatric patients with leukemia, silymarin intake improves liver function. Considering that free radicals resulting from lipid peroxidation are involved in various liver toxicities, the very strong antioxidant effect of silymarin may explain its protective effect on the liver.

## Data Availability

The raw data supporting the conclusion of this article will be made available by the authors, without undue reservation.
